# Tolerogenic Transcriptional Signatures of Steady-State and Pathogen-Induced Dendritic Cells

**DOI:** 10.3389/fimmu.2018.00333

**Published:** 2018-02-28

**Authors:** Emilia Vendelova, Diyaaeldin Ashour, Patrick Blank, Florian Erhard, Antoine-Emmanuel Saliba, Ulrich Kalinke, Manfred B. Lutz

**Affiliations:** ^1^Institute for Virology and Immunobiology, University of Würzburg, Würzburg, Germany; ^2^Institute for Experimental Infection Research, TWINCORE, Centre for Experimental and Clinical Infection Research, a joint venture between the Helmholtz Centre for Infection Research and the Hannover Medical School, Hannover, Germany; ^3^Helmholtz Institute for RNA-Based Infection Research (HIRI), Würzburg, Germany

**Keywords:** tolerogenic dendritic cells, transcriptional profiling, steady-state dendritic cells, bacteria, mycobacteria, helminths, immune evasion

## Abstract

Dendritic cells (DCs) are key directors of tolerogenic and immunogenic immune responses. During the steady state, DCs maintain T cell tolerance to self-antigens by multiple mechanisms including inducing anergy, deletion, and Treg activity. All of these mechanisms help to prevent autoimmune diseases or other hyperreactivities. Different DC subsets contribute to pathogen recognition by expression of different subsets of pattern recognition receptors, including Toll-like receptors or C-type lectins. In addition to the triggering of immune responses in infected hosts, most pathogens have evolved mechanisms for evasion of targeted responses. One such strategy is characterized by adopting the host’s T cell tolerance mechanisms. Understanding these tolerogenic mechanisms is of utmost importance for therapeutic approaches to treat immune pathologies, tumors and infections. Transcriptional profiling has developed into a potent tool for DC subset identification. Here, we review and compile pathogen-induced tolerogenic transcriptional signatures from mRNA profiling data of currently available bacterial- or helminth-induced transcriptional signatures. We compare them with signatures of tolerogenic steady-state DC subtypes to identify common and divergent strategies of pathogen induced immune evasion. Candidate molecules are discussed in detail. Our analysis provides further insights into tolerogenic DC signatures and their exploitation by different pathogens.

## Tolerogenic Dendritic Cells (DCs)

Tolerogenicity of DCs is an intrinsic functional definition for this cell type and their induction of T cell anergy, regulatory T cells and T cell deletion have been reported (1). All major DC subsets have been described to exert tolerogenic functions. Tolerogenic DCs were first described *ex vivo*, showing that UV-irradiated Langerhans cells induced T cell anergy (2). Spontaneous or UV-induced apoptotic cell death represents a source of self-antigens employed by DCs for tolerance induction. Steady-state mechanisms to maintain self-tolerance rely on the uptake of apoptotic material and its tolerogenic presentation (3–6). The ability to generate tolerogenic DCs *in vitro* facilitated their subsequent use for adoptive cell therapy in mice. However, *in vitro* generated immature DCs injected to protect from allo-transplant rejection matured, as indicated by their upregulation of B7-1 and B7-2 molecules, an unwanted phenomenon that was hypothesized to dampen the DCs tolerogenicity (7). Later, this hypothesis was confirmed by generating immature and maturation-resistant DCs in the same transplantation model, which dramatically extended the allograft survival time from 22 days to more than 120 days (8). Thus, maturation resistance was considered as a hallmark of tolerogenic DCs to maintain their immaturity. Several protocols have been developed to achieve maturation resistance, mostly using maturation inhibitors such as IL-10, TGF-β, dexamethasone, or vitamin D3 alone or in combinations (1). Reports on the transcriptional profiling of such DCs treated with tolerogenic substances followed and have been described elsewhere (9, 10).

Here, we analyzed transcriptional data sets deposited on public databases from steady-state migratory DCs (ssmDCs) and functionally similar spontaneously matured GM-CSF-derived bone marrow DCs (BM-DCs) as tolerogenic DC sources. Since ssmDCs act in a tolerogenic manner, despite their partial maturity, they phenotypically resemble much more mature DCs than non-migratory, immature DCs do. Therefore, they represent a more similar DC phenotype for our comparison of tolerance markers. We then analyzed transcriptional datasets of DCs treated with substances known to cause inflammation, including pathogen-derived molecules. The comparisons concentrated on bacteria or bacterial products but also included helminths, known as masters of immune evasion, but excluded protozoa and viruses. Candidate tolerogenic molecules that were highly upregulated by selected inflammatory or pathogenic stimuli in DCs are then discussed individually and compiled in tables.

## Tolerogenic Markers Identified for Steady-State and Pathogen-Exposed DCs

### Self-tolerance versus Microbial Immune Evasion

Dendritic cells residing in peripheral tissues at an immature stage act as immune sensors for pathogens. Pathogens, danger or inflammatory signals convert DCs into a mature/activated state which enables their migration into the draining lymph nodes. Subsequent stimulation of T cell immunity occurs by DC presentation of pathogen-derived antigens in the context of costimulation and proinflammatory cytokine production (11). In contrast, during homeostasis lymphoid organ-resident DCs and ssmDCs contribute to immune tolerance, thus controlling unwanted T cell responses against harmless or self-antigens (12).

Most microbes, especially those causing chronic infections, are evolutionarily well-adapted to their host. Such adaptation results in a balance between a pathogen-induced protective immune response and immune tolerance mechanisms that prevent microbial elimination. Infections with non-adapted microbes either kill the host rapidly or the microbe is immediately cleared by the host’s immune response. In both cases, the microbes cannot replicate and spread to another host. A successful microbe induces a chronic and preferably asymptomatic infection. This can be achieved by exploitation of the host’s immune tolerance mechanisms during pathogen–host coevolution.

Here, we analyzed public data in a comparative manner including tolerogenic and anti-inflammatory mRNA signatures of (1) steady-state DCs, (2) helminth-exposed DCs, (3) mycobacteria-exposed DCs, and (4) defined *in vitro* generated murine GM-CSF BM-DCs and human monocyte-derived DCs (MoDCs) treated with different inflammatory or pathogen-derived stimuli.

### Transcriptional Signatures of Tolerogenic Migratory DCs under Steady-State Conditions

**Figure 1:**
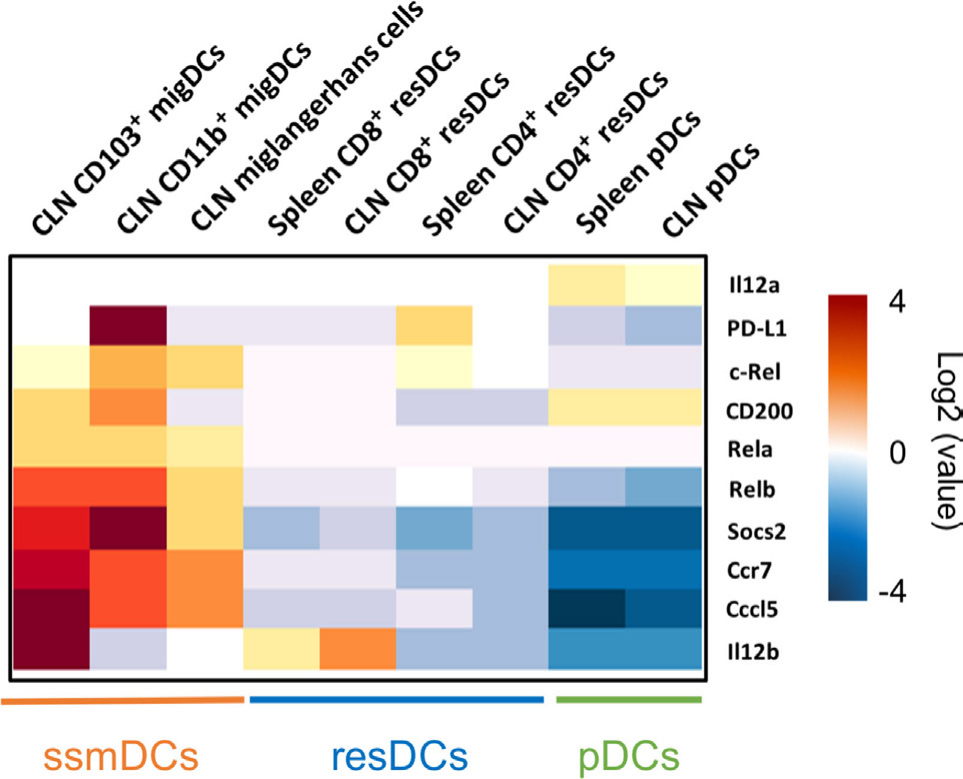
Major tolerogenic signatures upregulated on CCR7^+^ steady-state migratory dendritic cells (ssmDCs). Selected tolerogenic transcripts of *IL12b, RelB, Socs2, Cd200, Cd274*, and *Ccl5* are upregulated in CCR7^+^ ssmDCs (CD103^+^ migDC, CD11b^+^ migDC, Langerhans cells) as compared to CCR7^−^ resident DC subsets (CD8^+^ resDCs, CD4^+^ resDCs) or plasmacytoid DCs (pDCs). Data were obtained from the Immgen database (http://www.immgen.org/).

To identify tolerogenic DC signatures after pathogen stimulation, we first sought to identify comparative DC subsets known for their tolerogenic function as a reference dataset. While CCR7^−^ resident DCs appear at an immature stage, CCR7^+^ ssmDCs undergo a homeostatic maturation process reaching a semi-mature stage, which is characterized by low expression of MHC II and costimulatory molecules, such as CD40 and CD86, and the absence of proinflammatory cytokine production (13–16). In several respects, steady-state plasmacytoid DCs (pDCs) resemble resident CD4^+^ or CD8α^+^ conventional DCs (cDCs) of cutaneous lymph nodes and spleen (Figure 1). After pathogen-induced maturation DCs upregulate MHC II, CD40 and CD86 molecules on their surface (14, 15). Depending on the stimulus, mature RelB^+++^, RelA^+++^, and cRel^+++^ DCs differ qualitatively in the production of the proinflammatory cytokines IL-6, TNF, IL-1β, IL-12p70, IL-23, or type-I interferon, while RelB^+++^, RelA^+^, and cRel^+^ ssmDCs induce Tregs by their release active TGF-β^+^ from its latent form of surface-bound latency-associated peptide (LAP) molecules (14–16). While tolerogenic functions of ssmDCs have been described by many authors, the demonstration of T cell tolerogenicity by immature lymph node-resident DCs is much less understood (17). Thus, due to their increased maturity, we selected the tolerance markers of ssmDCs for comparison with pathogen-induced DCs.

In ssmDCs increased transcription of *Cd274 (*PD-L1), *CD200, Socs2, Relb, Ccl5*, and *IL12b* was observed as compared with pDCs and resDCs (Figure 1). Their enhanced transcription was observed in all three subsets of semimatured CCR7^+^ ssmDCs but not immature resident DCs of lymph nodes (Figure 1). In addition, high levels of *CD83, Cd150, Aldh1a2 (Raldh2,) Adora2a*, and *Itgb8* were found in ssmDCs (Table 1). Of these 11 molecules, 6 were also found in spontaneously matured BM-DCs (Table 1). The individual roles and mechanisms of tolerogenicity are explained below or referred to in Tables 1–5. Although the extent to which GM-CSF-derived BM-DCs resemble cDCs is still a matter for debate (18), the tolerogenic signatures observed in spontaneously matured BM-DCs (19) are strikingly similar to those observed in ssmDCs (14–16) (Table 1).

**Table 1:** Tolerogenic genes upregulated more than log2-fold by DCs matured during steady state, inflammation, or by pathogens.

Gene name	XCR1 + ssmDCs (15)	ssmDCs (14)	spont. mat. BM-DCs (19)	ImmGen data base	LPS (20)	LPS (21)	TNF (22)	LPS (22)	CT (23)	Mtb (24–26)	*Nippostrongylus brasiliensis*(27)	*Brugia malayi*(24)	*Schistosoma mansonii*(28)
*Il12b*	*Up*			*Up*	*Up*		*Up*	*Up*	*Up*		*Up*		*Up*
*RelB*	*Up*	*Up*		*Up*		*Up*	*Up*			*Up*	*Up*	*Up*	
*Ccl5*	*Up*			*Up*		*Up*					*Up*	*Up*	*Up*
*Socs2*	*Up*		*Up*	*Up*							*Up*		*Up*
*CD83*	*Up*		*Up*	*Up*	*Up*		*Up*			*Up*			
*Cd150 (Slamf1)*			*Up*	*Up*			*Up*	*Up*	*Up*		*Up*		
*Cd200*			*Up*	*Up*			*Up*				*Up*		*Up*
*Cd274*	*Up*		*Up*	*Up*							*Up*		
*Aldh1a2 (Raldh2)*			*Up*	*Up*			*Up*						
*Slamf7*							*Up*	*Up*	*Up*				
*Inhba*							*Up*	*Up*	*Up*				
*Ido1*	*Up*									*Up*			
*Adora2a*				*Up*				*Up*					
*IL-27*										*Up*	*Up*		
*Tgfb2*	*Up*												
*Itgb8*				*Up*									
*Optn*								*Up*					
*Thbs1*									*Up*				
*Vegfa*									*Up*				
*HLA-G*										*Up*			

*Steady-state migratory DCs/spontaneously matured BM-DCs*.

*LPS/TNF/CT-matured DCs*.

*Mycobacteria-matured DCs*.

*Helminth-matured DCs*.

### Tolerogenic Signatures of DCs Induced by Helminths

Due to evolutionary pressure, phylogenetically distinct parasitic worms—collectively termed “helminths”—convergently evolved the ability to manipulate their host’s immune systems. In nearly all cases, the antihelminth type 2 immunity of M2 macrophages and T helper cell 2 (Th2) cells fails to eliminate the worms (59, 60); hence helminths persist within their hosts for years. Helminths often exploit the host’s immune regulation machinery with DCs being major targets (59, 61, 62).

Type 2 immunity, in contrast to type 1, is promoted by weaker costimulation and/or absence of proinflammatory and polarizing cytokines such as IL-12p70 and IL-23 (13, 63). Moreover, the DC potential to induce type 2 immunity can be associated with tolerogenic mechanisms such as IL-10 secretion (63). Phenotypic maturation of DCs occurs after recognition of pathogen-associated molecular patterns (PAMPs) frequently inducing canonical NF-κB signaling (involving classical IκBα, -β, and -ε, NF-κB1 p50, RelA, and c-Rel). In contrast, recognition of helminths and their products by DCs results only in partial maturation resulting in low levels of costimulatory molecules at the surface and poor release of proinflammatory cytokines (64). It is believed that the non-canonical NF-κB pathway (Nfκb2/p52, RelB) not only direct cell development (65) but also might play a role in the regulation of immune tolerance (14, 66–68). Transcriptomic analyses of human DCs treated with Brugia malayi revealed upregulation of RELB and NFκB2 (24) and RelB in DCs isolated from mice after infection with *Nippostrongylus brasiliensis* (27) or *Schistosoma mansoni* eggs (28) (Table 1). This was similar to what has been observed in ssmDCs which induced Foxp3^+^ Tregs from naive T cells (14). In line with this hypothesis, Lacto-N-fucopentaose III, a carbohydrate found in *S. mansoni egg antigen*, has been shown to activate the alternative NF-κB pathway in DCs (69). Thus, non-canonical NF-κB activation in the absence of low activity of canonical RelA and cRel may be characteristic for tolerogenic DCs in helminth infections.

The activation status and cytokine release of DCs fine-tunes the polarization of different T cell-effector and regulatory mechanisms. Suppressor of cytokine signaling (SOCS) proteins play decisive roles in innate immune cell signaling. They modify the polarization of immune responses by negative regulation of cytokine signals (70, 71). Different helminth species promote upregulation of *Socs2* and *Socs3* (24, 27) (Table 1), which may skew immune responses toward a Th2-biased anti-inflammatory phenotype. Indeed, it was shown that SOCS3-transduced DCs express low levels of MHC II and CD86 molecules on their cell surface and produced high levels of IL-10 but low levels of proinflammatory cytokines such as IL12p70. They thereby induced Th2-cell differentiation in mice supporting allergic Th2 responses but impairing Th1/Th17 development by means of immune deviation toward Th2 as shown in the autoimmune model EAE (72, 73). As described above, tolerogenic ssmDCs express *Socs2* (Table 1). Therefore, induction of *Socs2* during helminth infection might even inhibit Th2 differentiation and instead support a tolerogenic environment (27, 74). It is not clear whether helminths induce *Socs* expression directly or through indirect cell mechanisms such as host-derived cytokines. For example, anti-inflammatory *Il27* is expressed in DCs after immunization with *Nippostrongylus brasilienis* (27) (Table 1). IL-27 induces expression of *Socs3* in mouse and human cells leading to induction of IL-10-producing Tr1 cells (75).

Different DC populations exposed to helminths induce expression of the regulatory cytokines *IL12b* and *IL-10* (27, 28). CD103^+^ migratory mature DCs from *N. brasiliensis* and *S. mansoni* infected mice significantly upregulate IL12b (27), also expressed in ssmDCs (Figure 1; Table 1).

Among others, *Cd200* and *Cd274* (PD-L1) were upregulated in DCs from *N. brasiliensis* immunized mice (Table 1). As detailed below, PD-L1 transmits inhibitory signals to PD-1 (CD279) on T cells. This interaction modifies TCR signaling, results in anergy or functional inactivation of T cells and is currently used for anticancer “checkpoint” inhibitory therapies (76, 77). PD-L1 expression would certainly support the chronicity of helminth infection. Suppression of T cell responses by PD1 during helminth infections has mainly been attributed to macrophages expressing PD-L1 and/or PD-L2 (78–80). Although the role of PD-L1 on DCs was not experimentally addressed, it may play a similar role.

Gene expression profiling using microarray or RNA sequencing technologies has been widely used to reveal cellular processes involved in host immune responses to different pathogens. Transcriptomic meta-analyses characterizing host immune responses against helminths have shown robust effects on immune gene signatures across different species (62). However, the common tolerogenic gene signature of DCs during helminth infection has not been addressed. Despite the fact that transcriptional profiling of DCs would improve our understanding of helminth effect during infection, the available helminth-related datasets are limited and further studies are required.

### Tolerogenic Markers Expressed after Infection with *Mycobacterium tuberculosis* (Mtb)

During coevolution with the human immune system, Mtb has developed multiple immune evasion strategies (81). To address whether Mtb is able to induce tolerogenic gene signatures in DCs, we analyzed transcriptional profiles of human DCs infected with Mtb and evaluated those for known tolerogenic markers.

Monocyte-derived DCs infected with Mtb or BCG highly upregulated the two tolerogenic genes *IDO-1* and *IL27*. *IDO-1* upregulation was detected already 8 h after infection of human MoDCs, whereas *IL27* transcripts were detected only upon Mtb, but not BCG, infection (25). Others showed upregulation of *RELB, CD83*, and *HLA-G* in MoDCs after 16 h of Mtb infection (24). The tolerogenic function of *RELB* is discussed below. *CD83* might also confer a regulatory function, as indicated by inhibition of T-cell proliferation that was mediated by the soluble form of the CD83 protein (58). Finally, HLA-G has been shown to induce human MoDC tolerogenicity *via* its CD85b/ILT4 ligand in huILT4-transgenic mice, inducing anergy and suppressor T cells (82). Hence, expression of *IDO, IL27, RELB, CD83*, and *HLA-G* (Table 1) by DCs might promote tolerogenic responses in Mtb infection.

### Tolerogenic Signatures of Murine and Human DCs Upregulated by Selected Inflammatory or Pathogenic Stimuli: TNF, Cholera Toxin, Lipopolysaccharide (LPS)

Transcriptional profiles of DCs stimulated *in vitro* under tolerogenic conditions have been reviewed before (10). Early transcriptional profiling work revealed that expression profiles of some cytokines are tightly regulated with time kinetic mRNA profiling revealing clear insights. IL-10 production stimulated by *Escherichia coli* LPS was only induced after 6 h in the DC cell line D1, but not earlier or later, whereas mRNA for TGF-β1 or IL-12p40 was detectable in time windows of more than 18–20 h after stimulation (83). DC cell line D1 showed IL-12p40 induction with LPS but not TNF (84) as reported for murine BM-DCs and human MoDCs (85). The fact that only two tolerogenic markers were identified in D1 cells may indicate a general limitation of obtaining transcriptional data from cell lines.

Of note, LPS stimulated DCs produce immunogenic Th1-polarizing IL-12p70, formed by the p35/p40 heterodimer (*Il12a* and *Il12b* genes), but the protein amounts of IL-12p40 secreted by DCs are typically 50–100 times higher than the amount measured for IL-12p70. Similarly, the IL-23 heterodimer secretion, composed of p19/p40 (*Il23a Il12b genes*) is much lower than p40 by cholera toxin stimulation of DCs (22, 23). This opens space for speculation on a counterbalancing and thereby tolerogenic role for excessive IL-12p40 production.

Dendritic cell maturation induced by inflammatory or microbial products triggering DAMPs or PAMPs, respectively, direct polarized Th1, Th2, or Th17 responses. Previously, we performed transcriptional profiling of murine GM-CSF generated BM-DCs and human MoDCs. Selected *in vitro* maturation protocols for induction of Th1 responses by LPS, Th2 by TNF and Th17 by cholera toxin (CT-DCs) were applied to both human and mouse DCs for the same time period of 6 h (22, 23) (GEO data bases GSE106080). Among the clearly immunogenic transcriptomic signatures, we also identified additional molecules at the protein level that exert tolerogenic immune functions. These include IL-10 production by LPS-DCs (86), Tr1 induction by Trypanosoma-matured or TNF-DCs after three injections (22, 87) and Foxp3^+^ Treg induction *via* TGF-β plus CTLA-2, a newly identified tolerogenic molecule from CT-DCs (23).

**Figure 2:**
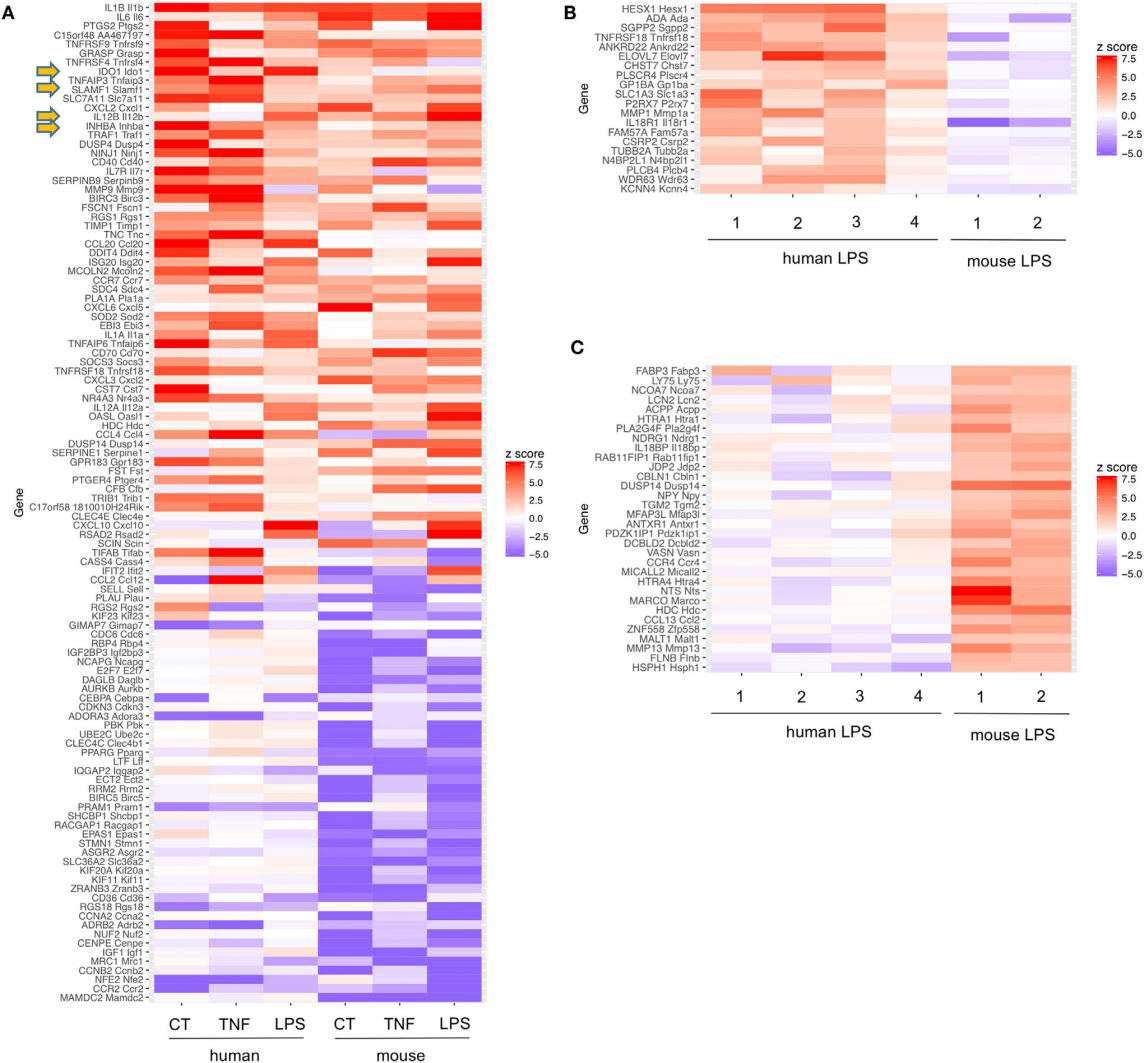
Common tolerance signatures of human monocyte-derived dendritic cells (MoDCs) or mouse bone marrow DCs (BM-DCs) stimulated with cholera toxin (CT), TNF, or lipopolysaccharide (LPS). **(A)** Among the top 14 genes upregulated under these conditions in DC of two different species, the 4 tolerogenic molecules *IDO, SLAM, Inhibin A*, and *IL12b* were found commonly upregulated (arrows). Expression signatures of strongly regulated genes in human and mouse DCs stimulated with CT, TNF, or LPS. Only genes with 1:1 ortholog mappings between human and mouse (obtained from MGI) were retained. *z*-scores were computed from the log2 fold changes for each experiment. Only genes having a *z*–score >4 or <−4 in at least two experiments are shown. Of note, for generation of human MoDCs additional IL-4 was added. Murine data are from Ref. (22, 23), human data obtained from GEO data bases (GSE106080). **(B,C)** Expression signatures of LPS stimulated human DCs [1 = (85); 2 = (88); 3 = (89); 4 = GSE106080] or mouse DCs [1 = (90); 2 = (22)]. Only genes with probe sets on each of the microarrays used were retained and *z*-scores were computed as in Panel **(A)**. In Panel **(B)**, genes with *z*-score > 2 in at least two human experiments and *z*-score < 0 in both mouse experiments are shown. Panel **(C)** depicts genes with *z*-score > 2 in both mouse experiments and <0 in at least two human experiments.

It remains a subject for debate whether the tolerogenic signature observed after infection has evolved as protective mechanism by the host or is actively induced by the pathogen. Pathogens aim to prevent their elimination and also the host aims to survive. If a pathogen cannot be eliminated, the host has to develop a protection strategy including the prevention of immunopathology. Excessive immune responses may be more deleterious than microbial pathogenicity in the host, as observed in sepsis. Thus, host-intrinsic negative feedback regulation of immune stimulation may be advantageous. To address this in our analyses, we included TNF as a non-pathogen-derived inflammatory stimulus. Interestingly, four tolerogenic genes showed increased mRNA transcription overlapping between TNF, CT and LPS stimulation (Figure 2) (Tables 1 and 2).

**Table 2:** Common transcripts induced under all six conditions (TNF, CT, LPS, each human and mouse data from Figure 2) and for which anti-inflammatory or tolerogenic functions have been reported.

Gene nameHuman/mouse, protein name	Functions	References for tolerogenicity
*IL12B/IL12b* IL-12p40	p40 homodimer antagonizes IL-12p70	(29, 30)
*IDO1/Ido1* Indoleamine-2,3-dioxigenase IDO	Metabolic inhibition of T cell proliferation by l-tryptophan catabolism	(31, 32)
*Cd150/SLAMF1/Slamf1* CD150/SLAM	Receptor for measles virus, inhibitor of DC function	(33–37)
*INHBA/Inhba* Inhibin βA, Activin βA	Partially inhibits DC maturation. Synergizes with TGF-β for induction of Foxp3^+^ Tregs	(38, 39)

Pathogens and inflammatory mediators induce numerous mechanisms of immunity in DCs. Additionally, molecules with tolerogenic or anti-inflammatory functions are induced. Mouse BM-DCs and human MoDC generated with GM-CSF (±IL-4) result from conversion of classical human CD14^+^ or mouse Ly-6C^high^ monocytes into DCs, in a human-mouse interspecies comparison. As expected, common proinflammatory genes such as *Il-1β, Il-6*, and *Cox2* (*Ptgs2*) were upregulated under all six conditions. Furthermore, four gene transcripts: *Il12b, Ido-1, Cd150* (*Slamf1*), and *Inhba* (coding for Inhibin/Activin) with reported anti-inflammatory or tolerogenic function were upregulated under all 6 conditions by stimulation with TNF, CT, or LPS of both human MoDCs or mouse BM-DCs (Figure 2 arrows, Table 2). Taken together, as these four genes were also upregulated by TNF, this tolerogenic DC response may reflect a host-initiated protection mechanism to avoid immunopathology rather than a purely pathogen-driven strategy.

Besides the common tolerogenic genes upregulated by all three stimuli, additional tolerogenic transcripts were found by the individual stimuli LPS (Table 3), CT (Table 4), and TNF (Table 5). These data indicate that microbial adaptation to the host and induction of tolerogenic signatures by LPS and CT not only share mechanisms of tolerogenicity but also differ in their strategies of immune evasion. Therfore, LPS selectively upregulates mRNA for adenosine A2a receptor, optineurin, and Slamf7/CD319, while CT induces higher transcription of thrombospondin-1 (TSP1) and Vegfa indicating divergent tolerance strategies (Tables 3 and 4).

**Table 3:** Tolerogenic transcripts induced specifically by LPS (human and mouse data from Figure 2) and for which anti-inflammatory or tolerogenic functions have been reported.

Gene nameHuman/mouse, protein name	Tolerogenic functions	References for tolerogenicity
*Adora2a*, Adenosine A2a receptor	Induces anti-inflammatory HO-1 production	(40)
*OPTN/Optn* Optineurin	Negative regulator of NF-κB	(41)
*SLAMF7/Slamf7* CD319	Immune cell inhibition	(42)

**Table 4:** Tolerogenic transcripts induced specifically by CT (human and mouse data from Figure 2) and for which anti-inflammatory or tolerogenic functions have been reported.

Gene nameHuman/mouse, protein name	Tolerogenic functions	References for tolerogenicity
*THBS1/Thbs1* Thrombospondin	Activator of latent TGF-β, regulator of DC cytokine production	(43, 44)
*VEGFA/Vegfa*	Suppression of DC differentiation and function	(45–48)

**Table 5:** Tolerogenic transcripts induced specifically by TNF (human and mouse data from Figure 2) and for which anti-inflammatory or tolerogenic functions have been reported.

Gene nameHuman/mouse, Protein name	Tolerogenic functions	References for tolerogenicity
*Cd200*	Immune regulatory in placenta, in pDC for IDO production and by pathogens	(49–51)
*ALDH1A2/Aldh1a2 (Raldh2)*	Coinducer with TGF-β or IL-4 for induction of Foxp3^+^ Tregs	(52, 53)
*RelB*	Expressed in self-antigen presenting, Treg inducing steady-state migratory DCs	(14, 16)
*CD83*	Secreted soluble CD83 induces Tregs, prevents T cell activation, and is highly tolerogenic in autoimmunity and allogeneic transplantation models	(54–58)

Since tolerogenic signatures of differentially stimulated human MoDCs and mouse BM-DCs were strikingly similar (Table 2), we asked whether also distinct differences exist between DC from the two species. Surprisingly, very few genes were selectively upregulated by human MoDCs but remained unaltered or downregulated in murine BM-DCs and vice versa (Figures 2B,C). Among those, no tolerogenic genes appeared. Interestingly, differences in the expression of *Gitr* (*Tnfrsf18*) were found, confirming known differences in expression and function of GITR in mice and humans on DCs (91). Thus, with respect to LPS sensing and transcriptional responses, human MoDCs and murine BM-DCs are remarkably similar.

## The Role of Selected Tolerogenic Molecules in Homeostasis and Immune Evasion

### Il12b

*Il12*b codes for IL-12p40 protein forming homo- and heterodimers. Two heterodimers can be formed with p40: p35/p40 that are linked *via* a disulfide bond to form IL-12p70 and p19/p40 to form IL-23. The release of IL-12p70 by DCs plays a pivotal role in the induction of Th1 responses (92, 93) while IL-23 supports Th17 generation (94, 95). However, the p40 monomer and especially the homodimer (p40)_2_ have been shown to strongly inhibit IL-12-dependent T cell or Th1 responses *in vitro* and *in vivo* (29, 30, 96), mainly by competing with IL-12p70. Interestingly, the total serum IL-12, and the ratio of IL-12p40/IL-12p70 increased with age in healthy individuals compared to IL-12p70 levels (97). This observation likely contributes to impaired immunity in the elderly. The expression of *IL12b* by ssmDCs is observed only in the CD103^+^ Langerin^+^ CD11b^low^ subset (15), and is significantly higher on ssmDCs when compared to lymphoid organ-resident DCs (Figure 1) (Table 1). Since *IL12a* mRNA coding for IL-12p35, is undetectable or at very low levels in any of the subsets under steady-state conditions, this may point to a tolerogenic role of p40 homodimers as described.

### Relb

RelB is an NF-κB/Rel transcription factor family member associated with both tolerogenic and immunogenic functions (98). The RelB-p50 heterodimer has been associated with inflammatory and immunogenic responses (68). In this case, it functions through the RelA-NF-κB canonical pathway and cooperates with the cRel-p50 heterodimer (65). cRel is specifically required for IL-12p70 production (99). On the other hand, the RelB-p52 heterodimer, which functions through the NF-κB non-canonical pathway, was shown to be important for organogenesis of lymphoid organs (100), for normal development of splenic CD4+ and CD8+ (101, 102) and ssmDCs (14). RelB, but absence of (or extremely low levels) of RelA or cRel, is expressed by migratory DCs both under steady-state conditions and upon immune activation (14, 15) (Figure 1). In the peripheral lymph nodes of p52−/− mice, the ssmDC subsets were severely reduced while the resident DCs were not affected. In contrast, p50−/− mice did not show a specific preference for migratory or resident DCs and both were equally reduced (14). Additionally, RelB-deficient animals show a severe pathological phenotype characterized by inflammatory infiltrates into multiple organs, which is caused by hyper activity of conventional T cells (100). RelB+ ssmDCs have been shown to be either critical for conversion of naive T cells into Foxp3+ iTreg (14, 103), or for maintaining the homeostatic Foxp3+ natural Treg pool (16). Together, the available data indicate that moderate RelB expression in DCs alone is associated with lymphoid organogenesis and tolerogenic functions, whereas coexpression of RelB with RelA and cRel at high levels in DCs marks immunogenic functions.

### CC Chemokine Ligand 5 (Ccl5)

The *Ccl5* gene encodes CCL5, also known as RANTES, has been described as a gene expressed by activated T cells, macrophages, eosinophils, fibroblasts, epithelial cells as well as certain types of tumor cells. CCL5 plays an important role in the migration of different leukocytes toward inflammatory sites where it acts through its binding to CCR1, CCR3, or CCR5 (104). One interesting observation is that certain types of tumors express high levels of CCL5, which is a predictor of a poor prognosis (105, 106). Blocking of CCL5 can redirect myeloid-derived suppressor cells (MDSCs) and thereby improve antitumor immunity (107). CCL5 has been shown to be important for the generation of CD11b^+^/Gr-1^+^ MDSCs and its absence alters their differentiation and their immunosuppressive capacity (108). CCL5 release by NKT cells was required for the recruitment of antigen-specific CD8^+^ regulatory T cells and TGFβ-dependent tolerogenic antigen-presenting cells in order to mediate tolerance in the immune-privileged anterior eye chamber (109). Given the higher *Ccl5* expression by ssmDCs relative to resident DCs it will be interesting to uncover its precise function in these cell types (Figure 1) (15).

### IL-10

Several TLR ligands, including LPS, induce IL-12p70 release from DCs to induce Th1 immunity and, in parallel, release of IL-10 (110). Listeria infection in neonates induces CD8α^+^ DCs to release IL-10 (111). The suppressive effect of IL-10 on Th1 responses is indirect *via* DCs or macrophages (112) and seems to control IFN-γ release but not proliferation of Th1 clones *in vitro* (113). This IL-10 production has been suggested to serve as a self-control mechanism to avoid Th1-mediated immunopathology (114) but also as a means of microbial immune evasion (115, 116). IL-10 can inhibit the differentiation of monocytes into Mo-DCs (117). Others found DC-to-DC effects by observing CpG-activated cDC-derived IL-10 blocked pDC release of type I interferons (118). Persistent production of IL-10 may then facilitate the conversion of Th1 (or Th2) responses into a IL-10^+^ Foxp3^−^ regulatory T cell response of the Tr1 type (119), similar to what had been observed for harmless antigen application and steady-state transport and Tr1 induction by lung DCs (120). The detailed regulation of IL-10 production (121) or its role of IL-10 for Tr1 cell induction has been reviewed elsewhere (122). However, although all this indicates an important role of IL-10 in immune tolerance, remarkably in none of the data sets analyzed herein (Figure 2; Table 1) was IL-10 identified as part of the tolerogenic transcriptional signature in DCs. The reasons for this may depend on delayed gene transcription kinetics or epigenetic regulation, thus identifying a limitation of tolerogenic transcriptional profiling.

### TGF-β/Itgb8

Foxp3 is the major transcription factor directing functions of thymus-derived natural Foxp3^+^ Tregs, but also peripherally induced Foxp3^+^ iTregs (123). Therefore, the production or employment of TGF-β by tolerogenic DCs for Treg generation or maintenance is of interest. TGF-β inhibits the maturation of BM-DC (124). However, murine BM-DCs produce soluble TGF-β when stimulated by *Lactobacilli* (125) and its release may be under the control of GITR (91). GM-CSF cultured BM-DCs lack the surface expression of LAP which can bind TGF-β in a latent form before it can be released for Treg induction (126). Therefore, they are unable to mediate iTreg conversion from naive CD4^+^ T cells *in vitro* without addition of exogenous TGF-β (23). In contrast, lymph node DCs express LAP and the partially matured ssmDCs do so at even higher levels when compared with immature resident DCs (14).

The release of active TGF-β from its latent form is the critical event in TGF-β biological activity. The integrins αVβ6 (Itgav, Itgb6) (127), αVβ8 (Itgav, Itgb8) (128), and *TSP1* (43) have been described to mediate non-proteolytic release of TGF-β, while metalloproteinase 9 (*MMP9*) performs proteolytic release (129). The activity of integrin αVβ8 has been shown as a key mechanism to prevent autoimmunity by maintaining Treg activity (130). Thus, these genes might be better markers for transcriptional signatures of TGF-β activity, although not identified in any of the RNA profiling data sets analyzed here (Figure 1). This indicates that not all important tolerogenic molecules are transcriptionally regulated and can be identified in such studies. A broader tolerogenic transcriptional signature was also identified for the subset of incompletely matured XCR1^+^ ssmDCs *ex vivo*, including the upregulation of TGF-β2 (15).

### Cd150/Slamf1

*Cd150* is upregulated on activated lymphoid and myeloid cells and acts *via* homotypic interaction (131). It represents the main human receptor for measles virus has been shown to inhibit DC functions (Table 2). Interestingly, the SH2D1A gene encoding for the SLAM-associated adapter protein to mediate SLAM signaling is mutated on X-linked immunodeficiency patients and responsible for the observed uncontrolled T and B lymphocyte proliferation after an EBV infection (132, 133). These data indicate that intact SLAM acts as an immune control molecule to prevent over activation of adaptive immunity during EBV infection.

### Indoleamine 2,3-dioxygenase (*Ido*)

IDO is an enzyme catabolizing l-tryptophan. Deprivation of this essential amino acid in the environment of proliferating T cells results in metabolic starvation, apoptosis and thus inhibition of the T cell responses (134). Interestingly, in pDCs a TGF-β-dependent tolerogenic function of IDO has been reported that is independent of its enzymatic activity (135). IDO also plays a decisive role in establishment of LPS tolerance *via* control of the aryl hydrocarbon receptor signaling (136).

### Inhba

The genes *INHBA/Inhba* encode for the Inhibin-βA or Activin-βA protein. Inhibin-βA forms homo- or heterodimers with other inhibin/activin family members to form the protein complexes Activin A (βA/βA homodimer), Inhibin B (α/βA heterodimer), or Inhibin AB (βA/βB heterodimer). They all belong to the TGFβ family (126) and many of the TGF-β family members influence DC development and function (137). Inhibition of DC maturation has been reported for Activin A and Inhibin A (38). Activin A may cooperate with TGF-β to increase generation of Foxp3^+^ induced regulatory T cells (iTregs) (39). Why Inhibin/Activin and not directly TGF-β are targets of immune evasion at the transcriptional level requires further investigation.

### Il27

IL-27 protein belongs to the IL-12 cytokine family and is a heterodimeric protein consisting of IL-27p28 and the Epstein-Barr virus-induced gene 3 (EBI3) (138). This cytokine is expressed early upon activation of antigen presenting cells. It has been shown to induce the initial step in Th-1 differentiation of naive CD4 T-cells by STAT1 dependent induction of T-bet (139). Besides this immunogenic function, several studies have analyzed the regulatory function of IL-27 during infection with various different pathogens (140). Infection with Mtb IL-27 was described to suppress T-cell responses by the reduction of TNF, IL-12p40, and IFN-γ expression and to inhibit T-cell recruitment and proliferation (141). Furthermore, IL-27 can induce the expression of IL-10 in activated CD4^+^ effector T-cells and thus reduce antimycobacterial activity (116).

### Socs2

Suppressor of cytokine signaling proteins play important roles in both the maintenance of homeostasis and the resolution of inflammation (71). Recent evidence suggests that SOCS2 plays a role in immune regulation. Similar to SOCS1 and SOCS3, also SOCS2 regulates pattern recognition receptor signaling in both human and murine DCs by counterregulating their activation (142). *Socs2*^−^*^/^*^−^ mice showed uncontrolled Th1 responses to *Toxoplasma gondii*, due to generalized proinflammatory responses to the infection (143). Besides innate immunity, SOCS proteins balance T helper cell polarization. SOCS1 and SOCS3 support Th17 cell generation by inhibiting Th1 differentiation while Th2 differentiation is regulated by SOCS3 (72, 73). SOCS2 was recently shown to play a major role in inhibiting the development of Th2 cells and Th2-associated allergic responses (74). However, whether SOCS expression in DCs is responsible for observed effects in T cells was not investigated by these studies. Here, we identified *Socs2* transcript elevation in all ssmDCs and spontaneously matured BM-DCs (Table 1) and upon *in vitro* exposure of DCs with *N. brasiliensis* and *S. mansoni*, further suggesting its important role in immune regulation (27, 28).

### Cd274

*Cd274* encodes programmed death ligand-1 (PD-L1) which delivers inhibitory signals *via* PD1 into T cells to regulate the delicate balance between immune defense and tissue-damage. PD-L1 is constitutively expressed or upregulated after activation on wide hematopoietic and non-hematopoietic cells and affect the responses against self and foreign antigens (76). Unsurprisingly, to evade immunity, microbes and tumors exploit the PD1/PD-L pathway which may act in concert with other immunosuppressive signals to establish chronic infection and tumor survival (76). Evidence that PD1/PD-L1 pathway is one of the main factors of tumor immune escape in humans is provided by the strategy of PD1/PD-L1 blockade. In addition to PD-L1 expression by tumors, myeloid DCs infiltrating tumors also express PD-L1. PD-L1 blockade improves myeloid DC-mediated antitumor immunity in several types of cancer (144). The blockade of this so called “check-point” has already been applied to clinical cancer therapy (145).

## Discussion and Future Perspectives by Single-Cell RNA-seq

The identification and the definition of DCs based on morphology, functional studies and surface markers have been subjected to many controversies and transcriptional studies have played a pivotal role in characterizing DC ontology (18). Disentangling DCs from monocytes and macrophages and understanding how DCs plasticity is shaped after stimulation or pathogen sensing remain technologically challenging because transcriptomics applied to a population of cells assumes a strict homogeneity among the cells, which does not reflect the biological reality. Genome-wide transcriptomics at the single-cell level (single-cell RNA-seq) is emerging as a powerful tool to phenotype cells and is elevating biased bulk approaches and profiling methods restricted to selected surface markers (146, 147). The revolution of single-cell RNA-seq lies in that cellular identities are no longer bounded by a restricted number of signals, but instead are inferred in an unbiased manner from an array of expressed genes. Single-cell RNA-seq can capture thousands of transcripts (148) to assess a cellular identity and enables profiling how a single-cell responds to stimulus. The response of *in vitro* differentiated DCs stimulated with three pathogenic components at the single-cell level (149, 150) revealed a dramatic difference between individual cells. The analysis demonstrated the existence of “gene modules” indicating the differential activation of gene circuits between cells where some cells are prone to mounting a precocious response, acting as “leaders” of an antiviral response. Furthermore, combining genome editing with CRISPR/Cas9-based technologies and single-cell RNA-seq helped to uncover the regulatory network controlling DC response to LPS (151). As a proof-of-concept the perturbation of *Rela, Irf9*, and *Cebpb* facilitated the decoupling of antiviral and inflammatory pathways. Such approaches, termed CRISPR-seq or Peturb-seq, are not only restricted to *in vitro* cultures, but can uncover the complexity of DC regulatory circuits *in vivo*. Notably, this approach has been used to resolve the contribution of STAT-1/2-dependent antiviral genes to myeloid cell function (151). Future applications of single-cell RNA-seq technologies should include in-depth studies of DCs exposed to pathogens, revealing their immunogenic and tolerogenic signatures.

## Conclusion

Activation-associated changes enabling DCs to activate adaptive immune responses are well understood. More recently, the scientific community has given greater attention to the counterregulation of these activation processes due to the clinical success of the checkpoint inhibitors, especially to the PD-1/PD-L1 molecules. Understanding of the tolerogenic mechanisms limiting inflammation is of utmost importance for therapeutic approaches that target immune pathologies, tumors and infections. As such, transcriptional profiling of tolerogenic DCs may provide insights into strategies allowing homeostasis and exploitation of own regulatory machinery by tumors and microbes.

Here, we aimed to uncover tolerogenic signatures of infla-matory or pathogen-matured DCs that included known tolerogenic markers of non-inflammatory ssmDCs. The present study addresses mainly transcriptomic studies as performed by microarray technologies of inflammatory or candidate bacteria- or helminth-induced DC signatures. This offered only a limited ability to fully identify all tolerance-associated mRNA species. However, our analysis revealed tolerogenic and anti-inflammatory genes among the expected expression of inflammatory genes. We reviewed the tolerogenic signatures of DCs exposed to different stimuli from both *in vitro* and *in vivo* studies across different host tissues and DCs subsets of man or mouse. Surprisingly, all pathogens analyzed here seem to use a rather restricted pool of target molecules for immune evasion. In the future, the possibility to quantify minute amounts of RNA species from single cells will enable analysis of much more complex regulatory networks in a wide variety of DC subsets.

## Author Contributions

All authors contributed by writing parts of the text and edited the final version of the text. Figures and tables were generated by EV, DA, FE, and ML.

## Conflict of Interest Statement

The authors declare that the research was conducted in the absence of any commercial or financial relationships that could be construed as a potential conflict of interest.
